# Supplementary material for: Nature of the spin resonance mode in CeCoIn5
Source: Commun Phys. Author manuscript; Available in PMC 2021 Mar 1. (PMC7919742; doi:10.1038/s42005-020-0365-8)
Supplement: Supp1 [file NIHMS1670547-supplement-Supp1.pdf]

Supplementary Materials:
Nature of the spin resonance mode in $CeCoIn_5$

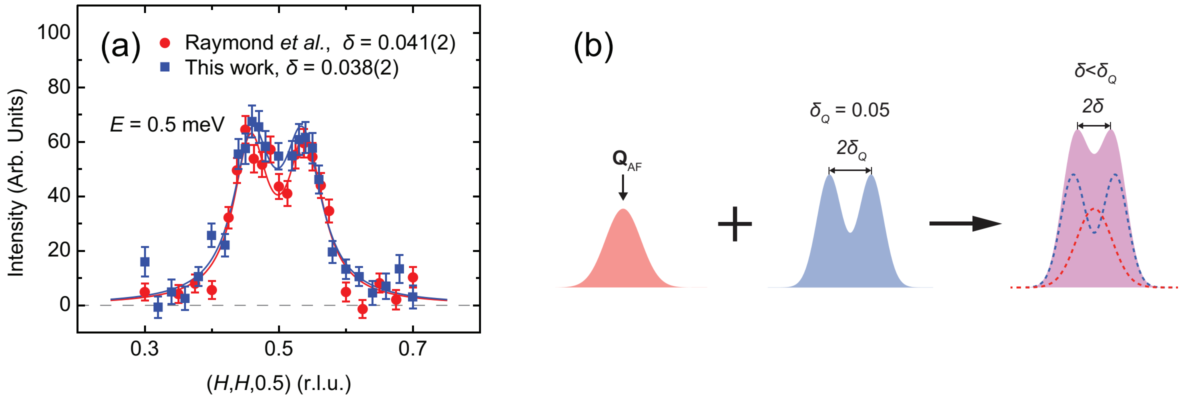

Supplementary Figure 1: Splitting of the SRM for $E \lesssim E_r$. (a) Comparison with previous report of incommensurate peaks for $E = 0.5$ meV in $CeCoIn_5$. The red squares are previously published results for $E = 0.5$ meV and $T = 1.45$ K [S1], and the blue circles are data for $E = 0.5$ meV and $T = 0.45$ K from Fig. 2(e) of the main text. The results are fit to two Lorentzian peaks at $\mathbf{Q} = (0.5 \pm \delta, 0.5 \pm \delta, 0.5)$ on linear backgrounds. The backgrounds have been subtracted and the data have been scaled to allow for a direct comparison. The obtained values of δ are in good agreement within uncertainty, and are significantly smaller than $\delta_Q = 0.05$ of the Q-phase. (b) Schematic of a possible scenario that accounts for the observed peak splitting δ being significantly smaller than δ_Q of the Q-phase.

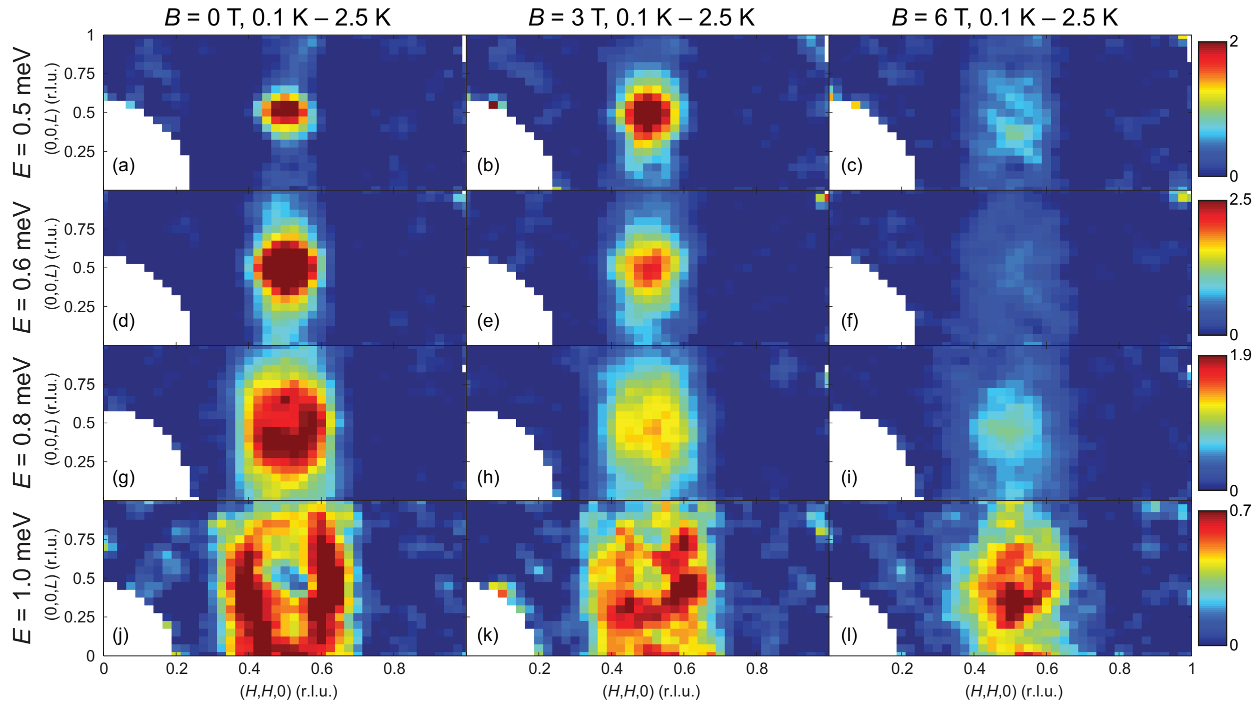

Supplementary Figure 2: $[H, H, L]$ maps measured using MACS, for (a)-(c) $E = 0.5$ meV, (d)-(f) $E = 0.6$ meV, (g)-(i) $E = 0.8$ meV and (j)-(l) $E = 1.0$ meV under magnetic fields $B = 0$, 3, and 6 T, respectively. The normal state response measured at $T = 2.5$ K has been subtracted. The data under $B = 0$ T are from Ref. [S2].

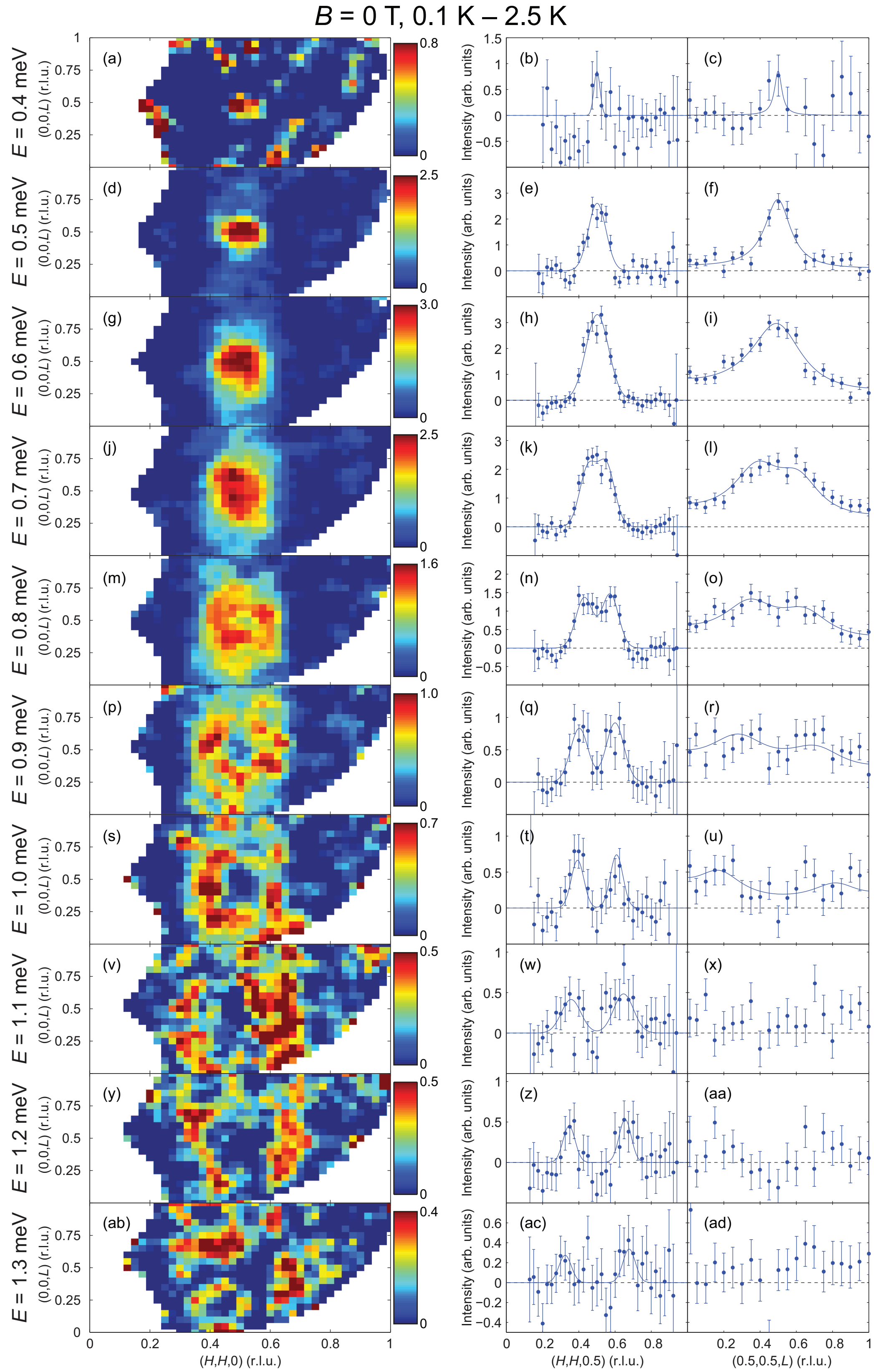

Supplementary Figure 3: $B = 0$ T $[H, H, L]$ maps measured using MACS, for (a) $E = 0.4$ meV, (d) $E = 0.5$ meV, (g) $E = 0.6$ meV, (j) $E = 0.7$ meV, (m) $E = 0.8$ meV, (p) $E = 0.9$ meV, (s) $E = 1.0$ meV, (v) $E = 1.1$ meV, (y) $E = 1.2$ meV and (ab) $E = 1.3$ meV. The corresponding cuts along $(H, H, 0.5)$ are respectively shown in (b), (e), (h), (k), (n), (q), (t), (w), (z) and (ac); the corresponding cuts along $(0.5, 0.5, L)$ are respectively shown in (c), (f), (i), (l), (o), (r), (u), (x), (aa) and (ad). The solid lines for $(H, H, 0.5)$ cuts are fits to a single Gaussian peak at $\mathbf{Q} = (0.5, 0.5, 0.5)$, or two Gaussian peaks at $\mathbf{Q} = (0.5 \pm \delta, 0.5 \pm \delta, 0.5)$; the solid lines for $(0.5, 0.5, L)$ cuts are fits to a lattice sum of a single Lorentzian peak at $\mathbf{Q} = (0.5, 0.5, 0.5)$, or two Lorentzian peaks at $\mathbf{Q} = (0.5, 0.5, 0.5 \pm \delta)$. The normal state response measured at $T = 2.5$ K has been subtracted.

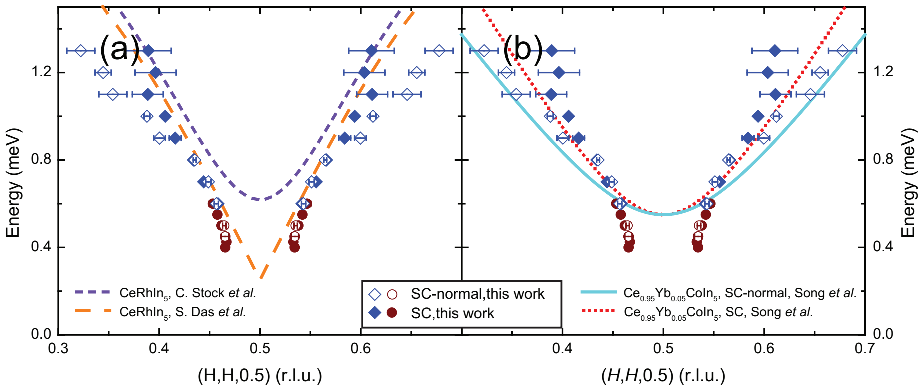

Supplementary Figure 4: Dispersion of the SRM in $CeCoIn_5$, compared with (a) spin waves in $CeRhIn_5$ [S3, S4] and (b) the SRM in $Ce_{0.95}Yb_{0.05}CoIn_5$ [S2]. The horizontal error bars are least-square fit errors (1 s.d.). Diamond symbols are from MACS data and circle symbols are from PANDA data. Dispersion of the SRM can be determined from the superconducting state response, with (open symbols) or without (solid symbols, also in Figs. 3(a) and (b) of the main text) subtracting the normal state response.

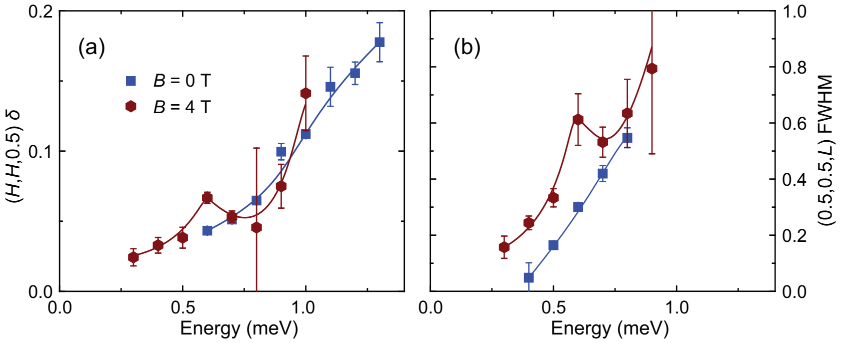

Supplementary Figure 5: Energy dependence of the peak splitting along $(H, H, 0.5)$ and the peak width along $(0.5, 0.5, L)$. (a) Energy dependence of the peak splitting δ obtained by fitting $(H, H, 0.5)$ cuts in Supplementary Fig. 3 and Supplementary Fig. 7 using two Gaussian peaks at $\mathbf{Q} = (0.5 \pm \delta, 0.5 \pm \delta, 0.5)$. (b) Energy dependence of the peak width by fitting $(0.5, 0.5, L)$ cuts in Supplementary Fig. 3 and Supplementary Fig. 7 using a lattice sum of Lorentzian peaks at $\mathbf{Q} = (0.5, 0.5, 0.5)$. The solid lines are guides-to-the-eye.

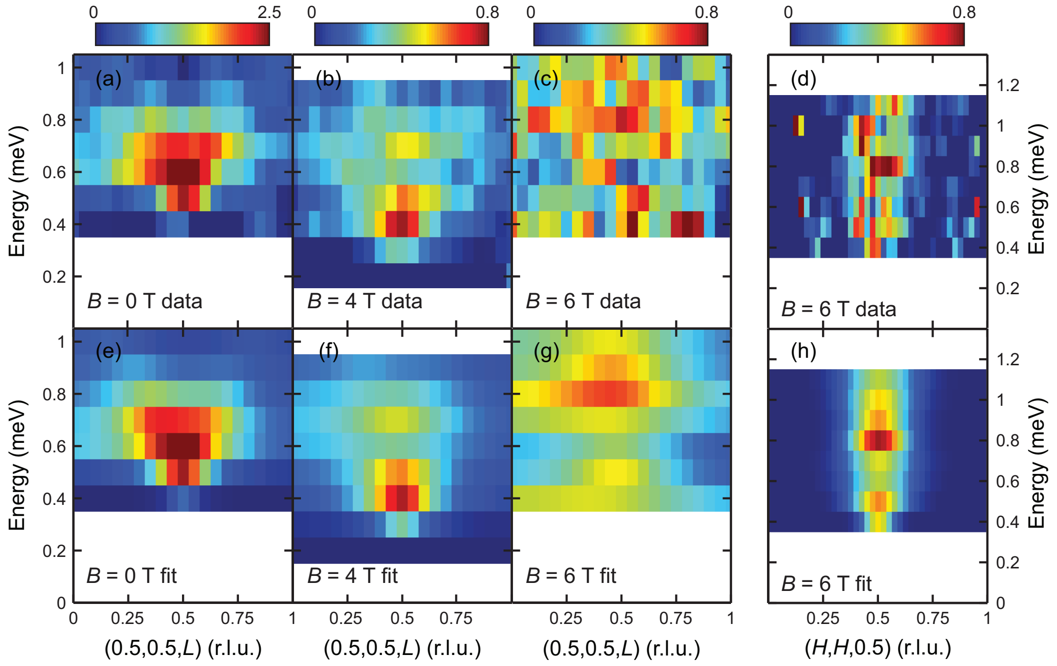

Supplementary Figure 6: Energy-$(0.5, 0.5, L)$ and energy-$(H, H, 0.5)$ maps of the SRM in $CeCoIn_5$ under applied magnetic field. Energy-$(0.5, 0.5, L)$ maps of the SRM in $CeCoIn_5$ for (a) $B = 0$ T, (b) $B = 4$ T and (c) $B = 6$ T, with the corresponding fits shown in (e), (f) and (g), respectively. (d) Energy-$(H, H, 0.5)$ maps of the SRM for 6 T, with the corresponding fits shown in (h), the 0 T and 4 T data are shown in Fig. 5 of the main text. The normal state response measured at $T = 2.5$ K has been subtracted. The fits are obtained by combining line fits in Supplementary Figs. 3, 7 and 8.

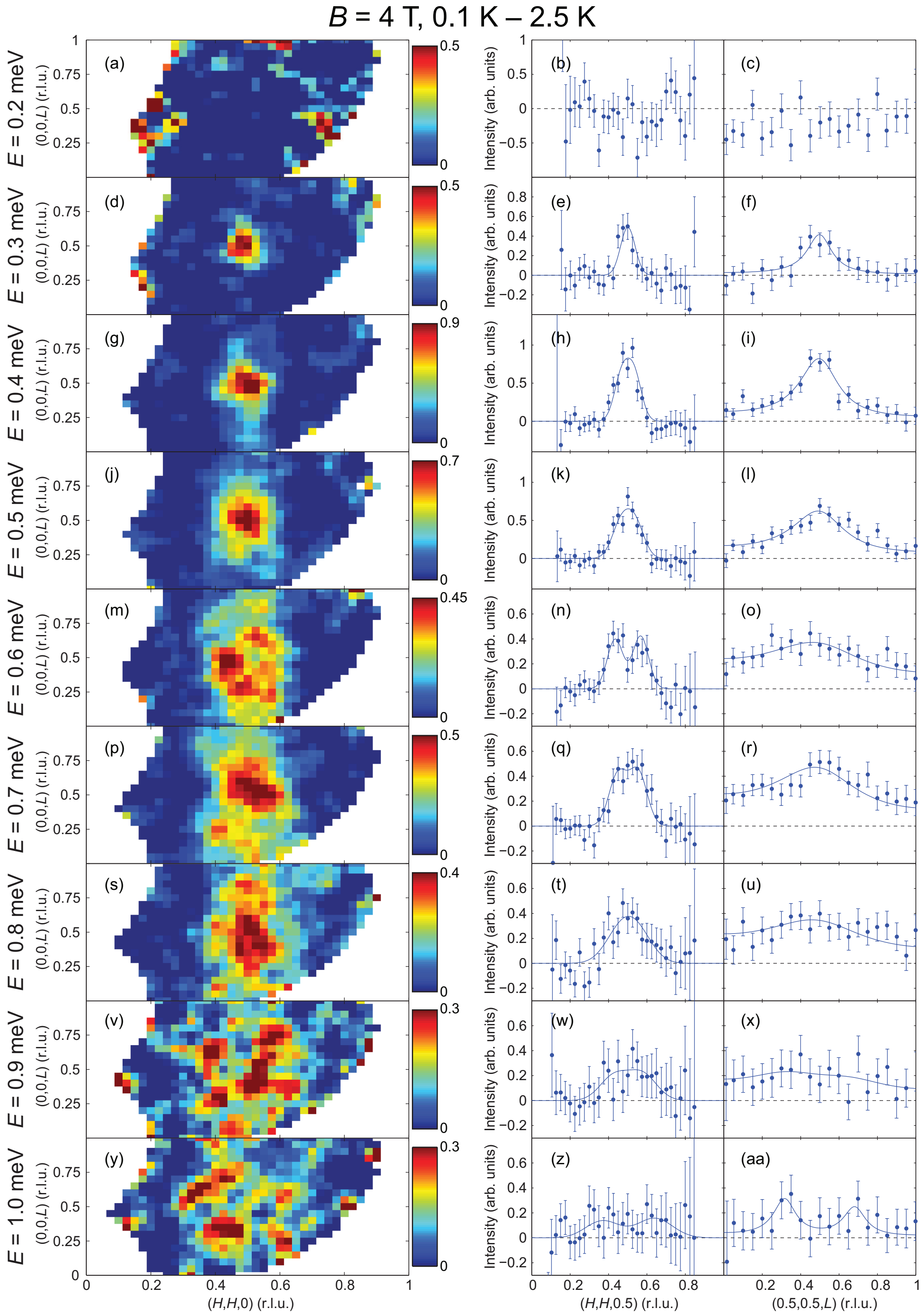

Supplementary Figure 7: $B = 4$ T $[H, H, L]$ maps measured using MACS, for (a) $E = 0.2$ meV, (d) $E = 0.3$ meV, (g) $E = 0.4$ meV, (j) $E = 0.5$ meV, (m) $E = 0.6$ meV, (p) $E = 0.7$ meV, (s) $E = 0.8$ meV, (v) $E = 0.9$ meV and (y) $E = 1.0$ meV. The corresponding cuts along $(H, H, 0.5)$ are respectively shown in (b), (e), (h), (k), (n), (q), (t), (w) and (z); the corresponding cuts along $(0.5, 0.5, L)$ are respectively shown in (c), (f), (i), (l), (o), (r), (u), (x) and (aa). The solid lines for $(H, H, 0.5)$ cuts are fits to a single Gaussian peak at $\mathbf{Q} = (0.5, 0.5, 0.5)$, or two Gaussian peaks at $\mathbf{Q} = (0.5 \pm \delta, 0.5 \pm \delta, 0.5)$; the solid lines for $(0.5, 0.5, L)$ cuts are fits to a lattice sum of a single Lorentzian peak at $\mathbf{Q} = (0.5, 0.5, 0.5)$, or two Lorentzian peaks at $\mathbf{Q} = (0.5, 0.5, 0.5 \pm \delta)$. The normal state response measured at $T = 2.5$ K has been subtracted.

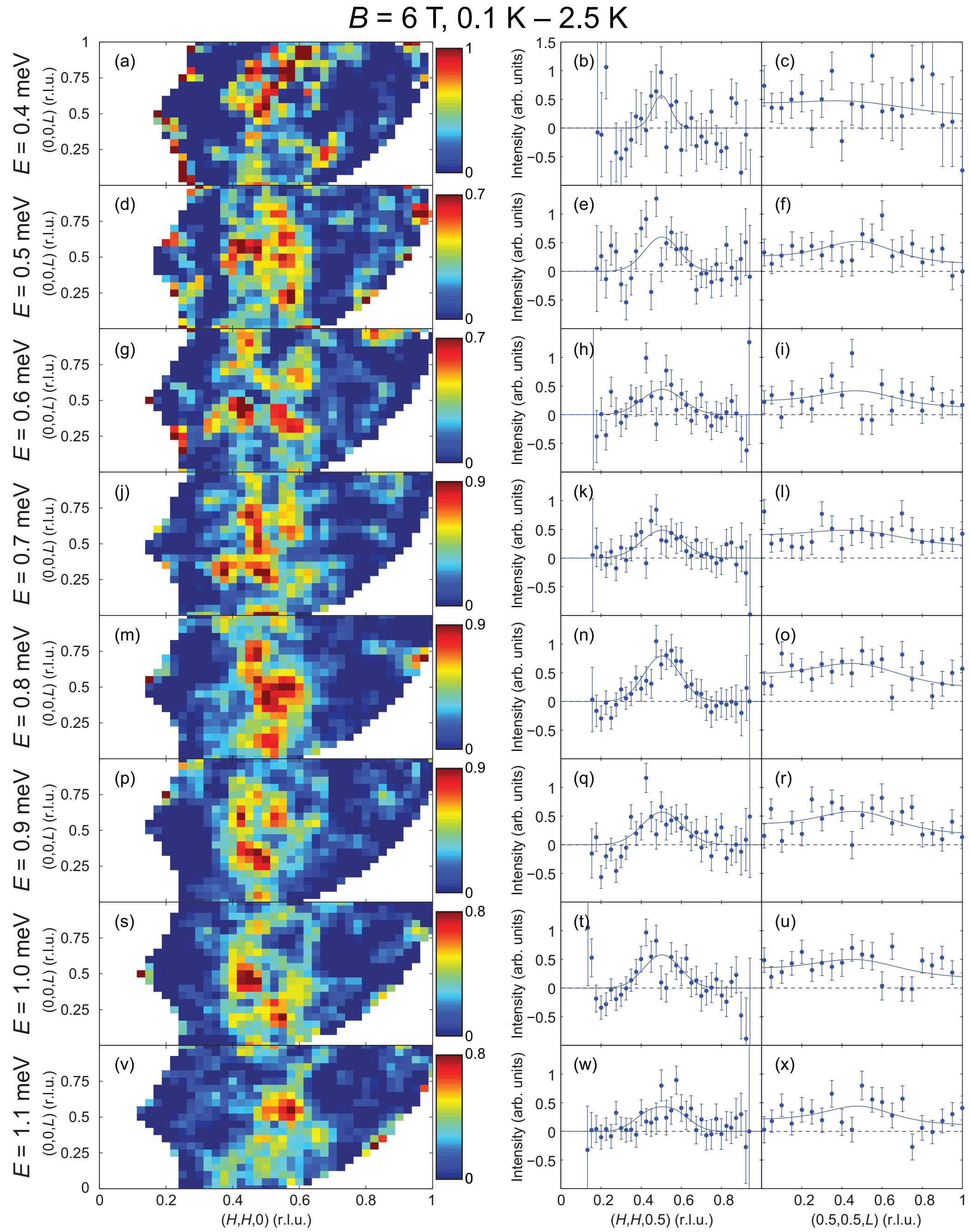

Supplementary Figure 8: $B = 6$ T $[H, H, L]$ maps measured using MACS, for (a) $E = 0.4$ meV, (d) $E = 0.5$ meV, (g) $E = 0.6$ meV, (j) $E = 0.7$ meV, (m) $E = 0.8$ meV, (p) $E = 0.9$ meV, (s) $E = 1.0$ meV and (v) $E = 1.1$ meV. The corresponding cuts along $(H, H, 0.5)$ are respectively shown in (b), (e), (h), (k), (n), (q), (t) and (w); the corresponding cuts along $(0.5, 0.5, L)$ are respectively shown in (c), (f), (i), (l), (o), (r), (u) and (x). The solid lines for $(H, H, 0.5)$ cuts are fits to a single Gaussian peak at $\mathbf{Q} = (0.5, 0.5, 0.5)$, or two Gaussian peaks at $\mathbf{Q} = (0.5 \pm \delta, 0.5 \pm \delta, 0.5)$; the solid lines for $(0.5, 0.5, L)$ cuts are fits to a lattice sum of a single Lorentzian peak at $\mathbf{Q} = (0.5, 0.5, 0.5)$, or two Lorentzian peaks at $\mathbf{Q} = (0.5, 0.5, 0.5 \pm \delta)$. The normal state response measured at $T = 2.5$ K has been subtracted.

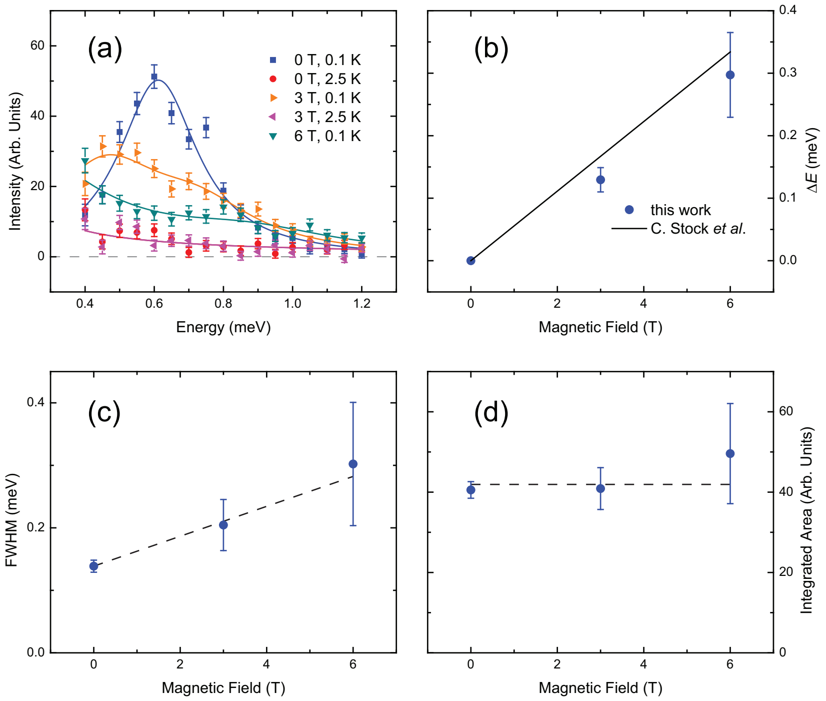

Supplementary Figure 9: Doublet splitting of the SRM at $\mathbf{Q}_{\mathrm{AF}}$ under applied field, measured using a single detector of MACS. (a) Constant-$\mathbf{Q}$ scans at $\mathbf{Q}_{\mathrm{AF}}$ under different experimental conditions. Solid lines are fits to the data as described in Supplementary Note 4. Background measured at 15 K has been subtracted from all scans. (b) Splitting ΔE of the SRM, with the form $E = E_0 \pm \Delta E$, as a function of magnetic field. The solid line is the splitting previously observed for $CeCoIn_5$ [S5]. (c) Full-width-at-half-maximum (FWHM) of the SRM as a function of applied field. The dashed line is a guide-to-the-eye. (d) Integrated area of the SRM as a function of applied field. The dashed line is a guide-to-the-eye.

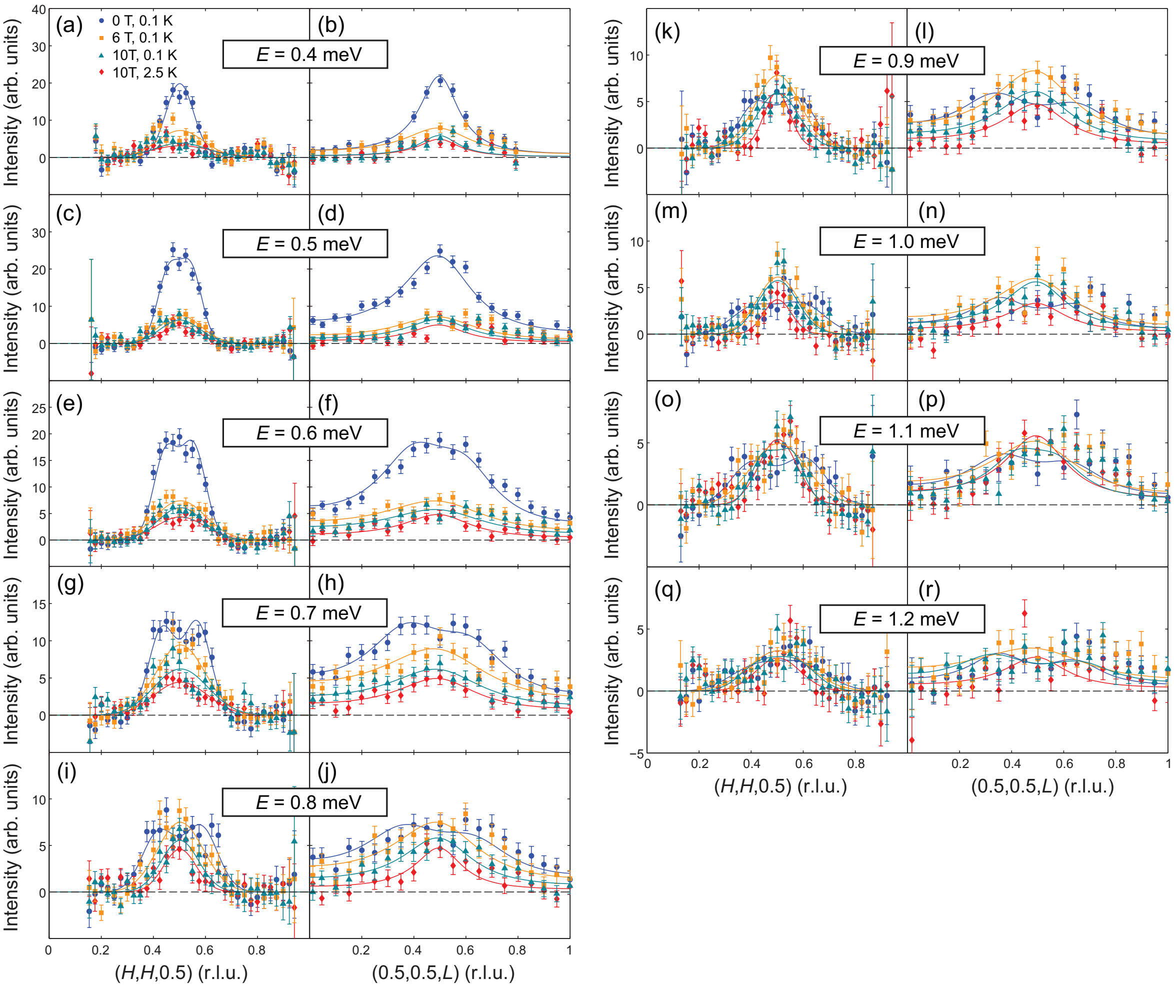

Supplementary Figure 10: Comparison of constant-energy cuts under different experimental conditions, obtained from $[H, H, L]$ maps measured using MACS. $(H, H, 0.5)$ cuts from $E = 0.4$ to 1.2 meV are shown in panels (a), (c), (e), (g), (i), (k), (m), (o) and (q). $(0.5, 0.5, L)$ cuts from $E = 0.4$ to 1.2 meV are shown in panels (b), (d), (f), (h), (j), (l), (n), (p) and (r). The solid lines for $(H, H, 0.5)$ cuts are fits to a single Gaussian peak at $\mathbf{Q} = (0.5, 0.5, 0.5)$ or two Gaussian peaks at $\mathbf{Q} = (0.5 \pm \delta, 0.5 \pm \delta, 0.5)$, plus an empirical $|\mathbf{Q}|$-dependent background; the solid lines for $(0.5, 0.5, L)$ cuts are fits to a lattice sum of a single Lorentzian peak at $\mathbf{Q} = (0.5, 0.5, 0.5)$ or two Lorentzian peaks at $\mathbf{Q} = (0.5, 0.5, 0.5 \pm \delta)$, plus an empirical $|\mathbf{Q}|$-dependent background. The empirical $|\mathbf{Q}|$-dependent background is constrained to be the same for $(H, H, 0.5)$ and $(0.5, 0.5, L)$ scans for each energy. In the plotted data and fits, the backgrounds obtained from fitting have been subtracted.

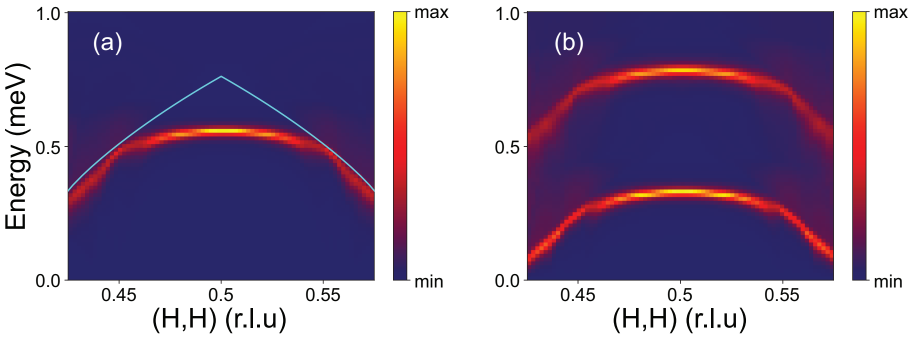

Supplementary Figure 11: Dispersion of the SRM in the spin-exciton scenario using $\bar{I}_0$ determined from scanning tunneling microscopy measurements [S6], in magnetic field (a) $B = 0$ T and (b) $B = 2$ T. The light blue line in (a) indicates the onset energy of the particle-hole continuum. See Supplementary Note 1 for details.

Supplementary Note 1: Spin-Exciton Scenario In Magnetic Field

In the spin-exciton scenario, the spin resonance mode (SRM) arises from a feedback effect of the sign-changing $d_{x^2-y^2}$-wave order parameter on the magnetic excitations in the system. To investigate this feedback effect, one computes the spin susceptibility in the random-phase approximation, given by

$$\chi(\mathbf{q},\omega) = \frac{1}{2}\frac{\chi_0(\mathbf{q},\omega)}{1+\bar{I}_0(\mathbf{Q})\chi_0(\mathbf{q},\omega)} \tag{1}$$

where χ_0 is the non-interacting susceptibility in the superconducting state and $\bar{I}_0(\mathbf{Q})$ is the magnetic interaction between f-electrons with momentum transfer $\mathbf{Q} = (\pi,\pi)$ (see Supplementary Information Section 3 of Ref. [S6] for details). As discussed in Ref. [S2], whereas the spin-exciton scenario predicts a downward dispersion of the SRM (see Supplementary Fig. 11(a), the light blue line indicates the onset energy of the particle-hole continuum, above which the SRM is strongly damped), experimentally a robust upward dispersion is observed, consistent with a magnon-like nature for the SRM.

As discussed in Ref. [S2], the splitting of the SRM under applied field into a doublet, rather than the triplet expected from a spin-1 excitation, can be understood as an effect of the system's magnetic anisotropy. The existence of a hard magnetic axis along the field direction ($[1\bar{1}0]$), with a corresponding easy plane perpendicular to it, leads to an anisotropy term in the magnetic Hamiltonian of the form

$$H_{\text{aniso}} = A\sum_{\mathbf{r}}(S^z_{\mathbf{r}})^2 \tag{2}$$

where $A > 0$ and the quantization axis z is taken along $[1\bar{1}0]$. This anisotropy changes the effective magnetic interaction for the longitudinal mode, $I^z_0(\mathbf{Q}) = I_0(\mathbf{Q}) + A$, relative to that of the transverse modes, $I^{\pm}_0(\mathbf{Q}) = I_0(\mathbf{Q})$. Since $I_0(\mathbf{Q}) < 0$, the anisotropy weakens the longitudinal magnetic interaction, pushing the corresponding SRM up to the onset of the particle-hole continuum. Thus, this mode is damped out and not observed experimentally.

We next want to consider the evolution of the SRM in a magnetic field. Given the above mentioned anisotropy, we consider only the transverse (to the magnetic field direction) spin modes $\chi^{\pm,\mp}$, which within RPA are given by

$$\chi^{\pm,\mp}(\mathbf{q},\omega) = \frac{\chi_0^{\pm,\mp}(\mathbf{q},\omega)}{1+\bar{I}_0(\mathbf{Q})\chi_0^{\pm,\mp}(\mathbf{q},\omega)} \,. \tag{3}$$

The expression for $\chi_0^{\pm,\mp}$ in a magnetic field [S7] is generalized to heavy quasiparticle bands of $CeCoIn_5$ as

$$\chi_0^{\pm,\mp}(\mathbf{q},\omega) = -\frac{1}{N}\sum_{\mathbf{k}}\sum_{i,j=\alpha,\beta}\zeta^2_{\mathbf{k+q},i}\zeta^2_{\mathbf{k},j}\left\{c^+_{ij}\frac{f^{\pm}_{\mathbf{k+q},i}-f^{\mp}_{\mathbf{k},j}}{\omega+i\delta+\xi^{\pm}_{\mathbf{k+q},i}-\xi^{\mp}_{\mathbf{k},j}} + \frac{c^-_{ij}}{2}\frac{1-f^{\mp}_{\mathbf{k+q},i}-f^{\mp}_{\mathbf{k},j}}{\omega+i\delta-\xi^{\mp}_{\mathbf{k+q},i}-\xi^{\mp}_{\mathbf{k},j}} - \frac{c^-_{ij}}{2}\frac{1-f^{\pm}_{\mathbf{k+q},i}-f^{\pm}_{\mathbf{k},j}}{\omega+i\delta+\xi^{\pm}_{\mathbf{k+q},i}+\xi^{\pm}_{\mathbf{k},j}}\right\} \tag{4}$$

with

$$\zeta^2_{\mathbf{k},i} = \begin{cases} w^2_{\mathbf{k}}, & i = \alpha \\ x^2_{\mathbf{k}}, & i = \beta \end{cases} \tag{5}$$

and

$$w^2_{\mathbf{k}},\ x^2_{\mathbf{k}} = \frac{1}{2}\left[1 \pm \frac{\left(\frac{\varepsilon^c_{\mathbf{k}}-\varepsilon^f_{\mathbf{k}}}{2}\right)^2}{\sqrt{\left(\frac{\varepsilon^c_{\mathbf{k}}-\varepsilon^f_{\mathbf{k}}}{2}\right)^2 + s^2_{\mathbf{k}}}}\right] \tag{6}$$

with $\varepsilon^c_{\mathbf{k}}$ and $\varepsilon^f_{\mathbf{k}}$ being the unhybridized conduction and f-electron dispersions, respectively, and $s_{\mathbf{k}}$ is the hybridization (see Supplementary Information Section 3 of Ref. [S6]). Here δ is the quasiparticle damping, $\xi^{\pm}_{\mathbf{k},i} = \Omega_{\mathbf{k},i} \pm g\mu_B SH$, $\Omega_{\mathbf{k},i} = \sqrt{E^2_{\mathbf{k},i} + \Delta^2_{\mathbf{k},i}}$, $c^{\pm}_{ij} = \frac{1}{2}(1 \pm \frac{E_{\mathbf{k}+\mathbf{q},i}E_{\mathbf{k},j}+\Delta_{\mathbf{k}+\mathbf{q},i}\Delta_{\mathbf{k},j}}{\Omega_{\mathbf{k}+\mathbf{q},i}\Omega_{\mathbf{k},j}})$, and $f^{\pm}_{\mathbf{k},i} = n_F(\xi^{\pm}_{\mathbf{k},i})$ is the Fermi function (the variables i, j ranging over the two quasiparticle bands α, β). The full spin susceptibility for the transverse modes in magnetic field is then given $\chi_{tot}(\mathbf{q},\omega) = \chi^{\pm}(\mathbf{q},\omega) + \chi^{\mp}(\mathbf{q},\omega)$.

The magnetic field leads to a shift in the superconducting dispersions by $\pm g\mu_{\mathrm{B}}SH$, where g is the effective g-factor, and S is the effective f-electron spin. Assuming $g = 2$ and $S = 1/2$, we show in Supplementary Fig. 11(b), the resulting dispersion of the resonance in a magnetic field with $H = 2$ T. Note that the downward energy shift of the lower mode is larger than the one observed experimentally, likely arising from the fact that the effective spin S is reduced due to Kondo screening or the effective Landé factor is reduced due to Ruderman-Kittel-Kasuya-Yosida interaction [S8]. Note that the energy of the SRM shifts in unison with the particle-hole continuum, such that the SRM remain essentially undamped. The latter observation is in disagreement with the experimental observations which suggest that the SRM and the particle-hole continuum move independently with increasing magnetic field, leading to the experimentally observed damping of the SRM (see Fig. 5 of the main text and additional discussion in Supplementary Note 4 on increased damping).

Supplementary Note 2: Comparison With Previous Results

Previously it was shown that the dispersion of the SRM in $Ce_{1-x}Yb_xCoIn_5$ resembles the dispersion of spin waves in $CeRhIn_5$ [S2]. Here we compare the dispersion of the SRM in $CeCoIn_5$ extracted from our PANDA and MACS data, with the spin waves in $CeRhIn_5$ [S3, S4], and the SRM in $Ce_{0.95}Yb_{0.05}CoIn_5$ [S2], in Supplementary Fig. 4. Dispersion of the SRM can be extracted either by fitting the superconducting state data (SC) or after subtracting the normal state response (SC-normal), in both cases the upward dispersion is clear and resembles the spin waves in $CeRhIn_5$ [Supplementary Fig. 4(a)]; dispersion of the SRM in $CeCoIn_5$ is slightly steeper compared to $Ce_{0.95}Yb_{0.05}CoIn_5$ [Supplementary Fig. 4(b)], suggesting that while the dispersive SRM remains robust upon Yb-doping, a small softening occurs.

Previously it was found that the SRM in $CeCoIn_5$ consists of two peaks at $\mathbf{Q} = (0.5 \pm \delta, 0.5 \pm \delta, 0.5)$ for $E \lesssim E_{\mathrm{r}}$, which was suggested to indicate the SRM is a precursor to the field-induced spin-density-wave in the Q-phase [S1]. In Supplementary Fig. 1 (a), incommensurate magnetic excitations at $E = 0.5$ meV found in previous [S1] and present work [Fig. 2(e) of the main text] are compared. The background-subtracted data are scaled and fit to two Lorentzian peaks (as done in Ref. [S1]), to allow for a direct comparison. These data agree very well, and both suggest the splitting of magnetic excitations at $E = 0.5$ meV to be significantly smaller than the splitting of incommensurate magnetic order in the Q-phase ($\delta_Q = 0.05$). As discussed in the main text, this discrepancy can be accounted for if the SRM for $E \lesssim E_{\mathrm{r}}$ consists of two contributions: one centered at $\mathbf{Q} = \mathbf{Q}_{\mathrm{AF}}$ and the other at $\mathbf{Q} = (0.5 \pm \delta_Q, 0.5 \pm \delta_Q, 0.5)$, as schematically depicted in Supplementary Fig. 1(b).

Supplementary Note 3: Detailed MACS Data

We have carried out comprehensive measurements of the SRM in $CeCoIn_5$ using the Multi-Axis Crystal Spectrometer (MACS) at the NIST Center for Neutron Research. All measurements other than those in Supplementary Fig. 9 were carried out by mapping the $[H, H, L]$ scattering plane using the 20 spectroscopic detectors. Cuts along $(H, H, 0.5)$ and $(0.5, 0.5, L)$ are then made from these data maps after folding the data into a single quadrant.

We have collected a high statistics data set, as shown in Supplementary Fig. 2, after subtracting measured normal state magnetic excitations at $T = 2.5$ K ($B = 0$ T). $(H, H, 0.5)$ and $(0.5, 0.5, L)$ cuts made from these maps are shown in Fig. 4 in the main text. Comparing the normal state excitations for $E = 0.8$ meV at $T = 2.5$ K, under applied fields of $B = 0$ T and $B = 3$ T (data not shown), we find there is no discernible field-dependence, therefore we conclude that the overdamped normal state excitations are unaffected or weakly affected by the applied magnetic field.

We also collected constant-energy maps with finer energy steps, as shown in the left panels of Supplementary Figs. 3 ($B = 0$ T), 7 ($B = 4$ T) and 8 ($B = 6$ T), after subtracting the normal state magnetic excitations measured at $T = 2.5$ K (under $B = 10$ T, here we assume the normal state excitations are independent of applied magnetic field, based on measurements discussed above). From the $(H, H, 0.5)$ and $(0.5, 0.5, L)$ cuts of these maps, shown in the right panels of Supplementary Figs. 3, 7 and 8, we constructed energy-$(H, H, 0.5)$ maps (Figs. 5(a) and (c) in the main text and Supplementary Fig. 6(d)) and energy-$(0.5, 0.5, L)$ maps (Supplementary Figs. 6(a)-(c)). The fits to these maps are obtained from the combination of fits to $(H, H, 0.5)$ and $(0.5, 0.5, L)$ cuts at each energy. The energy-$(0.5, 0.5, L)$ maps in Supplementary Fig. 6 reveals the SRM splits into a doublet under applied magnetic field, and becomes progressively damped with increasing field, consistent with findings from the energy-$(H, H, 0.5)$ maps.

We also carried out measurements under $B = 10$ T at $T = 0.1$ K, just below the upper critical field of $B_{c2} \approx 11.5$ T, and observed that the magnetic excitations are very similar to those in the normal state. $(H, H, 0.5)$ and $(0.5, 0.5, L)$ cuts measured under different experimental conditions (including $B = 10$ T) are compared in Supplementary Fig. 10, demonstrating damping to the magnetic excitations in the superconducting state progressively increase, approaching overdamped excitations in the normal state.

Supplementary Note 4: Increased Damping and Splitting Into Two Upward-Dispersing Branches Under Field

In addition to splitting into a doublet under applied magnetic field, the SRM in $CeCoIn_5$ also becomes progressively smeared out with field, as can be seen in Figs. 5(a) and (c) in the main text and Supplementary Fig. 6(d). These results suggest applied magnetic field also induces increased damping in $CeCoIn_5$. To directly study the effects of increased damping, we carried out constant-$\mathbf{Q}$ scans at $\mathbf{Q}_{\rm AF}$ under different applied fields using a single detector of MACS, with results shown in Supplementary Fig. 9 after a background measured at $T = 15$ K has been subtracted. The normal state responses are independent of applied field, and can be described by a Lorentzian response function. In the superconducting state, the sharp SRM at zero-field splits into a doublet and becomes broader in energy for $B = 3$ and 6 T. To quantify the evolution of the SRM with applied field, we fit the zero-field response to a damped harmonic oscillator

$$I(E) = \frac{A}{1 - e^{-\frac{E}{k_{\rm B}T}}} \frac{E\Gamma}{(E^2 - E_0^2)^2 + (2E\Gamma)^2}; \tag{7}$$

the $B = 3$ T and $B = 6$ T data are fit to two damped harmonic oscillators at $E = E_0 \pm \Delta E$

$$I(E) = \frac{A_1}{1 - e^{-\frac{E}{k_{\rm B}T}}} \frac{E\Gamma}{(E^2 - (E_0 - \Delta E)^2)^2 + (2E\Gamma)^2} + \frac{A_2}{1 - e^{-\frac{E}{k_{\rm B}T}}} \frac{E\Gamma}{(E^2 - (E_0 + \Delta E)^2)^2 + (2E\Gamma)^2}. \tag{8}$$

Under applied field, the lower branch of the doublet quickly moves out of our measured energy window, so a single damping parameter Γ is applied to both branches of the doublet; in addition, E_0 is constrained to be the same for $B = 0$, 3 and 6 T to extract ΔE. Size of the doublet splitting of the SRM under applied field ΔE is shown in Supplementary Fig. 9(b), consistent with previous work [S5]. The integrated area of the of the damped harmonic oscillator functions do not show a significant change up to $B = 6$ T, as shown in Supplementary Fig. 9(d). On the other hand, we find a clear increase in the full-width-at-half-maximum with field, as shown in Supplementary Fig. 9(c). This quantitatively demonstrates an increase in damping to the SRM under applied field, which accounts for the progressive smearing of dispersive features.

Based on Fig. 4 in the main text, we found the SRM splits into two upward dispersing branches under applied magnetic field. The same conclusion can be obtained by fitting the cuts in Supplementary Figs. 3 ($B = 0$ T) and 7 ($B = 4$ T). For $(H, H, 0.5)$ cuts, the peaks are fit with two Gaussian peaks at $\mathbf{Q} = (0.5 \pm \delta, 0.5 \pm \delta, 0.5)$, and fit values of δ are compared in Supplementary Fig. 5 (a). For $B = 0$ T, δ progressively increases with increasing energy, consistent with the zero-field SRM dispersing upwards. For $B = 4$ T, δ increases up to $E_{\rm r} \approx 0.6$ meV, decreases from 0.6 meV to 0.7 meV, and then increases from 0.7 meV to 1.0 meV. The non-monotonic evolution is consistent with the SRM splitting into two upward-dispersing branches. The increase of δ from 0.3 meV to 0.6 meV results from the upward dispersion of the lower branch, with little or no contribution from the upper branch; similarly, the increase in

δ from 0.7 meV to 1.0 meV is mainly due to the upward dispersion of the upper branch. The decrease of δ from 0.6 meV to 0.7 meV is because whereas 0.6 meV is dominated by the top of the lower branch, 0.7 meV is dominated by the bottom of the upper branch, therefore δ for 0.6 meV is significantly larger compared to 0.7 meV.

For $(0.5, 0.5, L)$ cuts, the peaks are fit with a single lattice sum of Lorentzian peaks centered at $L = 0.5$, and the widths of the Lorentzian peak are used to characterize the dispersion of the SRM along L, as shown in Supplementary Fig. 5 (b). Similar to the results from $(H, H, 0.5)$ cuts, for $B = 0$ T the width increases monotonically with increasing energy. On the other hand, for $B = 4$ T, with increasing energy the width first increases from 0.3 meV to 0.6 meV, then decreases from 0.6 meV to 0.7 meV, and finally increases from 0.7 meV to 0.9 meV. Such a non-monotonic evolution of the widths along L also supports the notion that the SRM splits into two upward-dispersing branches under applied magnetic field.

[S1] Raymond, S. & Lapertot, G. Ising incommensurate spin resonance of $CeCoIn_5$: a dynamical precursor of the Q phase. Phys. Rev. Lett. **115**, 037001 (2015).

[S2] Song, Y. *et al.* Robust upward dispersion of the neutron spin resonance in the heavy fermion superconductor $Ce_{1-x}Yb_xCoIn_5$, Nat. Commun. **7**, 12774 https://doi.org/10.1038/ncomms12774 (2016).

[S3] Das, P. *et al.* Magnitude of the Magnetic Exchange Interaction in the Heavy-Fermion Antiferromagnet $CeRhIn_5$, Phys. Rev. Lett. **113**, 246403 (2014).

[S4] Stock, C. *et al.* Single to Multiquasiparticle Excitations in the Itinerant Helical Magnet $CeRhIn_5$, Phys. Rev. Lett. **114**, 247005 (2015).

[S5] Stock, C. *et al.* Magnetic Field Splitting of the Spin Resonance in $CeCoIn_5$, Phys. Rev. Lett. **109**, 167207 (2012).

[S6] Van Dyke, J. *et al.* Direct evidence for a magnetic f-electron-mediated pairing mechanism of heavy-fermion superconductivity in $CeCoIn_5$, Proc. Natl. Acad. Sci. **111**, 11663-11667 (2014).

[S7] Ismer, J. P., Eremin, I., Rossi, E. & Morr, D. K. Magnetic Resonance in the Spin Excitation Spectrum of Electron-Doped Cuprate Superconductors. Phys. Rev. Lett. **99**, 047005 (2007).

[S8] Balcerzak, T. The RKKY interaction in the presence of an external magnetic field, Phys. Status Solidi C **3**, 212-215 (2006).
